# Measuring cognitive fusion through the Cognitive Fusion Questionnaire-7: Measurement invariance across non-clinical and clinical psychological samples

**DOI:** 10.1371/journal.pone.0246434

**Published:** 2021-02-03

**Authors:** Maria Anna Donati, Carmen Berrocal, Olivia Bernini, Costanza Gori, Caterina Primi

**Affiliations:** 1 Department of Neuroscience, Psychology, Drug, and Child’s Health, Section of Psychology, University of Florence, Florence, Italy; 2 Department of Surgical, Medical and Molecular Pathology, and Critical Care Medicine, University of Pisa, Pisa, Italy; 3 University Counselling Centre, University of Pisa, Pisa, Italy; University of Copenhagen, DENMARK

## Abstract

Cognitive fusion (CF) occurs when people are entangled in their private experiences. Rigid patterns of CF are a risk factor for various forms of psychopathology. The most widely used self-report instrument for assessing CF is the *Cognitive Fusion Questionnaire–7* (CFQ-7), a unidimensional scale with good reliability and validity. However, its psychometric properties have been studied mainly in non-clinical samples and by applying Classical Test Theory. The goal of this study was to use Item Response Theory to investigate the adequacy of the scale in a non-clinical sample and to test measurement invariance across non-clinical and clinical psychological samples. The non-clinical sample consisted of 258 undergraduate students (68.2% females, Mage = 24.3), while the clinical sample consisted of 105 undergraduate students with psychological distress (60.7% females, Mage = 23.8). The results showed that CFQ-7 assesses a wide range of CF severity among non-clinical subjects and that it is useful to discriminate different levels of CF. Moreover, the results showed the scale was sufficiently informative for a broad range of the trait. The relationships of CFQ-7 scores with theoretically related constructs provided further support to the validity of the scale. The Differential Item Functioning analysis showed that CFQ-7 is invariant across different types of population. Overall, findings in this study provide support for the adequacy of the CFQ-7 both in non-clinical and clinical contexts.

## Introduction

*Psychological flexibility* (PF) has emerged as an important construct to understand mental health and behavioral effectiveness [1–4]. PF has been described as “the ability to contact the present moment more fully as a conscious human being, and to change or persist in behavior when doing so serves valued ends” (p. 7) [2]. Acceptance and Commitment Therapy (ACT) is an evidence-based psychological intervention that was developed to promote PF [5]. According to the ACT model, various highly interrelated processes underlie PF, including cognitive defusion [2, 5].

Cognitive defusion is the ability to distance oneself from one’s thoughts and memories and to continue pursuing personal goals and values regardless of internal events one may be experiencing [2, 5]. In contrast, *cognitive fusion* (CF) occurs when people are entangled in, or dominated by, their private experiences [6]. In other words, CF occurs when behavior is guided more by thoughts and other internal experiences than by the direct experience with the world [2, 5]. Rigid patterns of CF have been proposed as a risk factor for various forms of psychopathology, since they may hamper PF [2, 5]. Indeed, high CF can exacerbate suffering (e.g., sadness, anxiety, anger, guilt), narrow behavioral repertoires and hinder effective actions for a meaningful life [2, 5]. CF with negative self-referential thoughts, such as ‘I am inadequate’ can elicit unpleasant mood states (e.g., sadness) that can make effective actions less probable and lead to the use of unhelpful avoidance strategies, such as worrying, rumination or thought suppression, to reduce discomfort [6]. Accumulated evidence suggests that these experiential avoidance strategies not only are ineffective but can also increase the frequency of unwanted internal experiences in the long run [7–9].

Gillanders et al. [10] developed the *Cognitive Fusion Questionnaire* (CFQ), a seven-item scale (CFQ-7), to have a general measure of CF that could be applied to different contexts. Indeed, the CFQ-7 has become the most widely used self-report instrument for assessing CF in both clinical and research settings [11]. It is a brief, free, and easy-to administer and easy-to-score measure. It is available in different languages, and its psychometric properties have been investigated across different countries and socio-cultural contexts [12–20]. Moreover, the CFQ-7 has been extensively exploited to address a range of research questions about CF and functioning in the fields of clinical and health psychology among clinical and non-clinical samples [11].

### Psychometric properties of the CFQ-7 among non-clinical samples

The psychometric properties of the CFQ-7 have been studied predominantly in non-clinical samples [10, 12, 18, 21]. Overall, the CFQ-7 has shown a one-factor structure [10, 12, 15, 18, 21, 22], very high internal consistency [12, 18, 21, 22] and adequate test-retest stability [10]. Additionally, the CFQ-7 has been shown to feature good criterion, convergent and incremental validity. Concerning criterion validity, moderate negative correlations have been found with PF [10, 12, 23], mindfulness [12, 15], committed action [12], quality of life and life satisfaction [12, 18, 21, 22], while significant positive relationships have been detected with both anxiety and depression [12, 15, 18, 21, 22]. As for convergent and incremental validity, previous research proved that the CFQ-7 strongly and positively correlates with psychological inflexibility [10, 15, 21], and that it significantly improves the predictive power of models for explaining psychological distress, above and beyond psychological inflexibility and thought suppression [21].

Despite such good evidence, the psychometric properties of the CFQ-7 have been analyzed so far only by using Classical Test Theory (CTT), without considering the possible application of Item Response Theory (IRT) models, which feature potential benefits in testing the accuracy in the assessment. Along with the discriminative power (*a*), IRT allows us to analyze item properties in terms of location, which can be conceptualized as the ‘severity’ of the phenomenon described by the item (*b* parameters). More specifically, IRT allows us to evaluate how well an item performs in measuring the underlying construct, the level of the construct targeted by the item and the appropriateness of the response categories [24]. Moreover, IRT indicates that measurement precision can vary at different levels of the trait. Hence, rather than providing a single value (e.g., an alpha coefficient) for reliability [25, 26], the precision of the test is assessed at different levels of the measured construct via the Test Information Function (TIF).

Another advantage of IRT is latent trait estimation. In IRT, latent trait scores can be estimated by using the model parameter estimates by searching for values that maximize the likelihood of observed patterns of responses to all the items in the test [27]. Specifically, we used IRT trait estimates for the CFQ-7 to analyze the validity of the scale. Applied research showed that the IRT summed-score approach is a valid method than can be applied for various research purposes (e.g., [28, 29]), as in the evaluation of test validity (e.g., [30]).

Following these premises, the first step in our study was to apply IRT models to investigate the psychometric properties of the CFQ-7 in a sample of a non-clinical population, consistent with several previous studies [10, 12, 18, 21]. We initially aimed at confirming unidimensionality and then at analyzing the characteristics of the items in terms of severity and discrimination, as well as the accuracy of the scale along the continuum of the trait with the TIF.

After that, the criterion validity of the scale was investigated to obtain evidence about the accuracy of the CFQ-7 in measuring CF, taking into account committed action, depression, and life satisfaction. Committed action refers to actions that are linked to and guided by goals and values [2, 6, 31]. Committed actions are further characterized by being persistent and flexible–that is, they continue to be taken even when they trigger difficult internal experiences, and they are discontinued when unsuccessful [6, 31]. According to the ACT model, committed actions generate vitality; hence, they are essential for a meaningful life and to prevent excessive suffering [2, 6]. Furthermore, committed action and CF are conceptualized as two interrelated processes, both contributing to PF [2, 6]. Indeed, findings from various studies demonstrated negative correlations between committed action and CF [15, 32, 33]. Accordingly, in this study we hypothesized that higher CFQ-7 scores would correlate negatively with committed action and life satisfaction, and positively with depression.

### The CFQ-7 across non-clinical and clinical samples

A critical issue concerns the functioning of the CFQ-7 across different populations, specifically, clinical and non-clinical. In their developmental study, Gillanders et al. [10] obtained the final seven-item version of the CFQ through explorative factor analyses conducted with university samples. They also analyzed some of the psychometric properties in medical and psychological clinical populations. Consistently, various studies have analyzed the psychometric properties of the CFQ-7 in clinical samples. In particular, unidimensionality, good internal consistency, concurrent and convergent validity have been found in individuals with chronic pain [15], in participants of a program aimed at enhancing stress management abilities, in patients with different mental health problems, in patients with multiple sclerosis [10] and in people with other medical problems, such as osteoarticular disease, diabetes, and obesity, or with psychological difficulties, such as major depression [14]. Furthermore, previous research about the incremental validity of the CFQ-7 in clinical populations showed that the scale explains incremental variance in distress, beyond psychological inflexibility, in prison officers; in depression, beyond positive beliefs about rumination and ruminative response style, in depressed, recovered, and never depressed subjects; and, in distress, beyond helplessness beliefs and psychological inflexibility, in patients with sclerosis [10].

Some studies have also investigated measurement invariance of the CFQ-7 across non-clinical and clinical samples in a CTT framework. Gillanders et al. [10] concluded that factor loadings and error covariances were not invariant across five different non-clinical and clinical samples due to significant Δ*χ^2^* values between the baseline and the constrained model. By fixing the factor loadings across samples, Ruiz et al. [22] found the CFQ-7 to be invariant for all the criteria recommended by Cheung and Rensvold [34] and Chen [35]. However, both studies compared only the unconstrained model and the model with equality constraints on factor loadings. Instead, Costa et al. [14] tested more levels of invariance (i.e., from configural invariance to structural invariance), and their results supported strict measurement invariance and structural invariance of the CFQ-7 across five different groups (four clinical groups and one non-clinical group). Thus, only a few partly contrasting findings support measurement invariance of the CFQ-7 across the two kinds of groups.

The capacity of an instrument to function effectively in different groups of respondents is a fundamental prerequisite to test differences across the groups. It allows us to ascertain whether the detected differences relate to group membership and not to the measured construct (that is, whether a measure is biased because respondents who belong to different groups but hold the same characteristics with respect to the measured construct, answer differently). Hence, measurement invariance of the CFQ-7 should be more deeply analyzed in order to interpret whether differences in CF across different types of samples–for example, higher CF scores in patients with psychological/psychiatric difficulties [10, 22] and with major depression [14] than in non-clinical samples–reflect real differences in CF. Indeed, invariance ensures both the fairness and the validity of group comparisons when examining a specific psychological construct [36].

Item Response Theory allows for the assessment of measurement invariance in terms of Differential Item Functioning (DIF) [25], which examines whether the likelihood of endorsing each item is equal across subgroups that are matched on the measured trait. For instance, if a test contains items with non-significant DIF across different samples (e.g., non-clinical vs clinical), one can assume that a randomly selected person from the non-clinical sample with a certain level of the underlying construct (named trait level of *θ*) and a randomly selected person from the clinical sample with the same level of *θ* should have the same likelihood of endorsing a particular response option for each item on the scale.

Our second aim was to test measurement invariance across samples through DIF analyses in an IRT framework, which is considered to be one of the most efficient methods for analyzing a test’s measurement invariance [37]. After this necessary step, we investigated differences in CF across samples, by hypothesizing that the clinical group would obtain higher CFQ-7 scores than the non-clinical group.

Summing up, the goal of our study was to investigate the psychometric adequacy of the CFQ-7 in an IRT framework. First, with a non-clinical sample, we aimed at confirming unidimensionality, and at verifying the item properties in terms of severity and discrimination, and the accuracy of the entire test along the trait. Moreover, we were interested in exploring the criterion validity of the scale by testing the relationships of IRT trait estimates for the CFQ-7 and theoretically related variables (committed action, depression, and life satisfaction). Then, having verified measurement invariance of the CFQ-7 across samples through DIF analyses, we investigated differences across samples concerning CF.

## Materials and methods

### Participants

The study included a non-clinical and a clinical sample of college students from the University of Pisa (Italy). Participants were recruited by convenience sampling. The non-clinical sample consisted of 258 undergraduate students enrolled in psychology and medical courses. Most participants were females (68.2%) and their ages ranged from 18 to 58 years (*M* = 24.3; *SD* = 10.0).

Concerning the clinical sample, we used baseline data from ongoing broader research aimed at comparing the effectiveness of an ACT-based intervention with a traditional Cognitive-Behavioural treatment among students seeking assistance at the counselling centre of the University of Pisa. The centre provides free counseling services for undergraduate students who apply for help with a broad range of social and emotional concerns, such as exam anxiety, relationship difficulties, poor concentration and mood disturbances. To participate in the study, students had to be at least 18 years old, speak Italian fluently and show mild to moderate depressive and/or anxiety symptoms according to the *Hospital Anxiety and Depression Scale* (HADS > 3 on anxiety or depression subscales, and HADS < 15 on both anxiety and depression subscales) [38].

A total of 107 undergraduate students applying for psychological intervention at the counselling centre participated in the study. They were mostly female (60.7%) and their ages ranged from 19 to 35 (*M* = 23.82; *SD* = 3.04). According to HADS total scores, most students (n = 101, 94.4%) were probably clinical cases (HADS ≥ 11), a total of five students (4.7%) were probably borderline cases (7 < HADS < 11) and only one participant (0.9%) yielded a ‘normal’ HADS total score (HADS ≤ 7).

### Measures

The research protocol included a form for collecting socio-demographic information (age, gender), the CFQ-7, and self-report measures of committed action, depression and life satisfaction.

The CFQ-7 [10] consists of seven items which are rated on a Likert scale from 1 to 7. Higher CFQ-7 total scores indicate higher cognitive fusion. Examples of CFQ items are: ‘My thoughts cause me distress or emotional pain’ and ‘I tend to get very entangled in my thoughts’. The Italian version of the CFQ-7 is shown in S1 Appendix. The scale demonstrated high internal consistency in the non-clinical sample (Cronbach alpha coefficient = .88).

The 18-item version of the *Committed Action Questionnaire* (CAQ-18) [31], Italian version by Donati et al. [39], was used to measure actions connected to goals and values (i.e., committed action). Items are rated on a Likert-type scale ranging from 0 to 6, with higher scores indicating greater levels of committed action. Examples of CAQ-18 items are ‘I am able to pursue my goals both when this feels easy and when it feels difficult’ and ‘If I feel distressed or discouraged, I let my commitments slide’. The questionnaire showed high internal consistency (α = .91) [31] and concurrent validity with different measures of functioning, such as depression, social functioning, vitality and general health) [31, 40, 41]. Previous research also showed that CAQ-18 scores correlated in the expected direction with other established components of psychological flexibility, such as acceptance, CF and mindfulness abilities, supporting the construct validity of the scale [31, 32, 40, 41]. In this study, the Cronbach alpha coefficient in the non-clinical sample was .88, and descriptive indices suggested that the total score (*M* = 89.05, *SD* = 15.03, range = 51–133) had a normal distribution (skewness = .11, kurtosis = -.04).

The *Beck Depression Inventory-I* (BDI–I) [42], Italian version by Scilligo [43], was used to assess depression phenomenology. It consists of 21 items rated on an ordinal scale from 0 to 3. Higher scores on the BDI-I indicate higher depression severity. The questionnaire demonstrated high internal consistency, with mean alpha values of .86 and .81 for clinical and non-clinical samples, respectively, and adequate concurrent validity with respect to other measures of depression [44]. The Cronbach alpha coefficient in the non-clinical sample was .85, and the total score (*M* = 9.59, *SD* = 7.52, range = 0–45) had a non-normal distribution (skewness = 1.48, kurtosis = 3.25).

The *Satisfaction with Life Scale* (SWLS) [45], Italian version by Di Fabio and Palazzeschi [46], is a five-item scale designed to measure global satisfaction with life regardless of emotional states. Items are scored on a scale from 1 to 7. Higher SWLS scores indicate greater satisfaction with life. The SWLS consistently demonstrated high internal consistency (alpha coefficients ranging from .79 to .89), and it showed moderate to high correlations with other indices of subjective well-being and with measures of distress [47]. The Cronbach alpha coefficient in the non-clinical sample was .87, and descriptive indices suggested that the total score (*M* = 22.29, *SD* = 6.08, range = 51–133) had a normal distribution (skewness = -.43, kurtosis = -.63).

### Procedure

This study was approved by the institutional review board of the University of Pisa. A back-translation design, according to the guidelines of the International Test Commission [48], was used to translate the English version of the CFQ-7 into Italian. First, a native Italian speaker, who is fluent in English, translated the CFQ-7 into Italian. The resulting version of the CFQ-7 was then translated back into English by a second translator who is a native speaker of English and fluent in Italian, and who has an in-depth knowledge of the Italian culture. Two Italian-speaking researchers, who are fluent in English and have knowledge of the ACT model, resolved semantic discrepancies between the original version and the back translation, and produced a second Italian proof of the scale. This new version of the CFQ-7 was then translated back into English and, again, the researchers resolved discrepancies in respect to the original version in order to enhance, as much as possible, the linguistic equivalence of the final Italian version.

Students received a form providing information on the study characteristics, and they provided written informed consent prior to being included in the study. Participation was voluntary and anonymous and had no effect on the students’ academic standing. To control potential ordering effects, the questionnaires were sorted and presented according to five different sequences which were randomly generated.

Participants in the non-clinical group were recruited during class time using opportunity sampling from various medical and psychology university courses. They completed a paper-and-pencil version of the survey. Participants in the clinical group were recruited among students that consecutively applied for psychological intervention at the counselling centre. To access the service, students had to request a first appointment via an online booking system and to complete an online version of the HADS. Students meeting HADS inclusion criteria then attended an assessment session conducted by a psychologist who provided information about the study, evaluated the remaining inclusion criteria and invited selected participants to complete an online baseline survey, which included the CFQ-7.

### Statistical analyses

The IRT analyses were conducted using IRTPRO 2.0 [49] and, according to the CFQ-7 response format, Samejima’s [50] Graded Response Model (GRM) was used. First, we verified the key assumptions on the data postulated by this model: unidimensionality, local independence and suitability of the IRT model for the data [51]. In terms of unidimensionality, we checked the distribution of the items for assessment of normality, and then the factor structure of the CFQ-7 was tested using Confirmatory Factor Analysis (CFA) in order to demonstrate that the scale measures a single latent construct (trait or *θ*). The CFA was conducted with AMOS 16.0 [52], using maximum likelihood estimation on the variance-covariance matrix. The local independence was assessed using the *χ*^2^ LD statistic [53], which is computed by comparing observed and expected frequencies in each of the two-way cross tabulations between responses to each pair of items. Since this diagnostic statistic is approximately distributed as standardized *χ*^2^, values of 10 or greater indicate the presence of local dependence. To verify that the IRT model fits the data, we then used the M_2_ statistic and the associated RMSEA value. RMSEA values of .05 or less indicate a good fit [54]. The item fit under the GRM was then tested by computing for each item the S—*χ*^2^ statistics [55]. Significant S—*χ*^2^ statistics indicate that the item did not fit under the model [56]. Given that using larger samples often leads to a greater likelihood of significant chi-square differences, the critical value of .01 rather than the usual critical value of .05 was employed [57].

We then analyzed the item properties. IRT models estimate probabilities of responses as a function of *θ*—that is, a continuous variable with a mean conventionally fixed at 0 and an *SD* of 1.0. For this model, logistic curves, called category response curves (CRCs), are generated for each response option of each item, showing the probability of a response to the option as a function of the underlying trait. Thus, for each item, threshold parameters (β_i_) equal to the number of response options minus 1, are derived indicating the trait level, where there is a 0.5 probability of endorsing the relevant response option or higher response options. Values can be interpreted as the ‘intensity’ of the phenomenon described by the item; therefore, the higher the level of the trait on which the threshold values are located, the higher intensity of the item referred to for the latent construct.

Additionally, the GRM provides one discrimination parameter (α), which refers to the ability of an item to discriminate among respondents with different levels of *θ*. Thus, an item is expected to have high levels of *a* (discrimination), and *b*s (severity) that are evenly spaced along the trait, as it means that the item categories provide an adequate differentiation in measuring *θ*.

The next step was to analyze reliability. IRT makes it possible to assess how precise the test is via the TIF, which evaluates the precision of the test at different levels of *θ*. The more information (*I*) the test provides at a particular level of the underlying trait, the smaller the error associated with the trait estimation, and the higher the test’s reliability. The associated reliability is 1 minus the inverse of the information the test provides [*r* = 1 - (1/*I*)]. The TIF basically shows how accurately the construct is measured at different levels of *θ*. To study the criterion validity of the scale, first we calculated IRT estimate scores, which allowed us to estimate the trait level of each respondent simultaneously with the item parameters [58]. IRT estimate scores were computed with the expected a posteriori (EAP) estimation method [25, 59], which is an excellent computational option for unidimensional scales [60]. In particular, EAP estimation computes the mean of the posterior distribution of *θ*, given the observed response pattern [25, 59].

Analyses of DIF across samples were then performed by applying the IRT Likelihood Ratio test approach (IRTLR) [61] via IRTPRO [49]. This procedure involves comparing differences in log-likelihoods (distributed as chi-square) associated with nested models. Since DIF analyses examine differences in item parameters, two types of DIF can be detected in the GRM model: uniform DIF (for the location parameters) and non-uniform DIF (for the discrimination parameter). Finally, we explored the differences in CF, as measured through the CFQ-7, across the non-clinical and clinical sample. This procedure involved comparing differences in log-likelihoods (distributed as chi square) associated with nested models. Because multiple tests were performed, the level of significance of .05 was adjusted by Bonferroni correction to .003 (.05/14).

## Results

Univariate distributions of the CFQ-7 items were examined for assessment of normality [62]. All items showed a normal distribution, with skewness values ranging from -.04 to .46 and kurtosis indices ranging from -1.01 to -.51 (Table 1). Then, the unidimensional structure was tested by a CFA. Goodness of fit indices were all adequate (CFI = .983, TLI = .974, RMSEA = .060). Standardized factor loadings ranged from .61 to .78, and they were all significant at the .001 level (Table 1). Moreover, none of the LD statistics was greater than 10.

**Table 1:** Skewness, kurtosis, fit statistics, standardized factor loadings, item discrimination, and category threshold estimates (with the standard errors in brackets) of the seven items of the *Cognitive Fusion Questionnaire-7* (CFQ-7) in the non-clinical sample.

Item	Sk	Ku	*λ*	S—*χ*^2^(*df*)	*p*	*a* (*SE*)	*b*_1_ (*SE*)	*b*_2_ (*SE*)	*b*_3_ (*SE*)	*b*_4_ (*SE*)	*b*_5_ (*SE*)	*b*_6_ (*SE*)
**1**	.04	-.61	.78	83.51 (68)	.097	2.39 (.26)	-1.78 (.17)	-.69 (.10)	-.21 (.09)	.82 (.11)	1.67 (.16)	2.63 (.28)
**2**	.46	-.51	.77	72.72 (61)	.144	2.46 (.27)	-1.29 (.13)	-.13 (.09)	.39 (.09)	1.21 (.13)	2.12 (.21)	2.90 (.35)
**3**	-.04	-.86	.65	121.26 (92)	.022	1.59 (.18)	-2.38 (.27)	-1.17 (.15)	-.52 (.12)	.20 (.11)	1.12 (.15)	1.89 (.21)
**4**	.18	-.85	.71	74.76 (80)	.645	2.14 (.23)	-1.44 (.14)	-.61 (.10)	.06 (.09)	.69 (.11)	1.41 (.16)	2.20 (.23)
**5**	.08	-.81	.61	105.40 (88)	.099	1.44 (.17)	-1.74 (.21)	-.85 (.14)	-.24 (.11)	.69 (.14)	1.70 (.21)	2.47 (.30)
**6**	.01	-.91	.78	65.11 (71)	.675	2.48 (.28)	-1.79 (.16)	-.79 (.10)	-.18 (.09)	.54 (.10)	1.15 (.13)	2.56 (.28)
**7**	.01	-1.01	.70	82.70 (85)	.551	1.88 (.21)	-1.69 (.18)	-.81 (.12)	-.24 (.10)	.41 (.10)	1.13 (.13)	2.07 (.21)

Note. Likert scale ranges from 1 = ‘Never True’ to 7 = ‘Always True’. Sk = Skewness, Ku = Kurtosis, λ = standardized factor loadings, df = degrees of freedom, a = item discrimination, bi = category threshold estimates, SE = standard error.

After verifying the unidimensionality of the scale, we conducted unidimensional IRT analyses. Samejima’s [50] GRM model was tested, and the fit statistics indicated an adequate fit (M2 = 1141.02, *df* = 749, *p* < 0.001; RMSEA = .05). The level of significance of .05 was adjusted by Bonferroni correction to .003 (.05/14). Each item showed a non-significant S—*χ^2^* value (Table 1), thereby indicating that all the items fit the graded unidimensional model.

We then looked at the item parameter estimates (Table 1). According to Baker and Kim’s [63] criteria, discrimination parameter values were high for most of the items, especially for items 2 and 6. The item parameters covered a broad range of the trait, i.e., from about 1.50 *SD* below the mean to about 2.50 *SD*s above the mean value. Item 3 showed *b* parameters located in lower regions of the trait in comparison with the other items.

S1 Fig shows the CRCs for each item. Each CRC indicated that there was a good separation in the response options and that the curves of each response option were distributed across the trait range.

**Fig 1:**
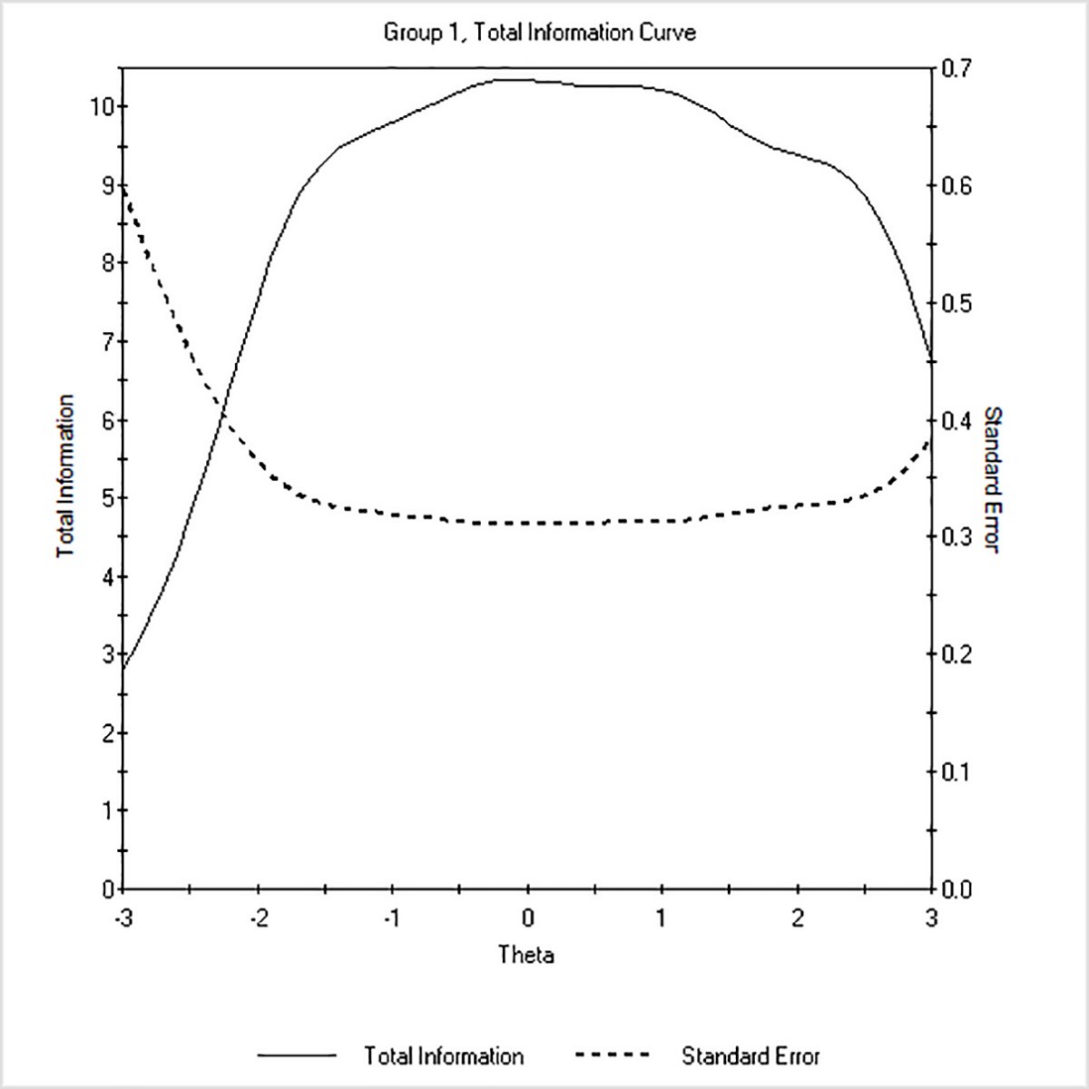
Test Information Function (TIF) of the *Cognitive Fusion Questionnaire-7* (CFQ-7) under the Graded Response Model (GRM) in the non-clinical sample (n = 258). Latent trait (*θ*) is shown on the horizontal axis, and the amount of information and the standard error yielded by the test at any trait level are shown on the vertical axis.

Concerning reliability, the TIF indicated that the scale was sufficiently informative for a broad range of the trait (Fig 1). Test information ranged from about -2.50 *SD*s to about +3.00 *SD*s. Considering that *θ* distribution in the sample ranged from -2.60 to +3.19, the scale was informative in correspondence to the sample scores. Moreover, the amount of test information was ≥ 4, with values ≥ 9 starting from a trait level of -1.50 to +2.00, corresponding to *r* of about .90 for this range of the trait.

Next, we analyzed the criterion validity of the CFQ-7 by using *θ* scores. As shown in Table 2, as expected, CFQ-7 *θ* scores were significantly and negatively correlated with both committed action and life satisfaction, while they were significantly and positively correlated with depression. The results also showed that committed action was negatively correlated with depression and positively correlated with life satisfaction.

**Table 2:** Descriptive statistics and correlations between CFQ-7 total scores and measures of committed action, depression, and life satisfaction in the non-clinical sample (n = 258).

	1	2	3	4
**1 CFQ-7 *θ* scores**	-			
**2 CAQ-18 summed scores**	-. 47***	-		
**3 BDI-I summed scores**	.59***	-.42***	-	
**4 SWLS summed scores**	-.42***	.49***	-.51***	-
***M***	-.01	89.05	9.59	22.29
***SD***	.95	15.03	7.52	6.08

*** *p* < .001. CFQ-7: *Cognitive Fusion Questionnaire-7*; CAQ-18: *Committed and Action Questionnaire-18*; BDI-I: *Beck Depression Inventory;*

SWLS: *Satisfaction with Life Scale*.

After finding that the CFQ-7 had an adequate functioning in the non-clinical sample, DIF analyses were conducted using the non-clinical sample (*n* = 258) as the reference group, and the clinical sample (*n* = 107) as the focal group. The results showed from the first step that no item showed DIF. Item DIF statistics ranged from .0 to 1.1 for the discrimination parameters, with associated *p*-values ranging from .29 to .91 and from 3.9 to 13.6 for the threshold parameters, with associated *p*-values ranging from .03 to .69 (Table 3). Thus, the CFQ-7 can be considered invariant across types of population.

**Table 3:** Differential Item Functioning (DIF) of discrimination and severity parameters across non-clinical and clinical samples for the seven items of the *Cognitive Fusion Questionnaire-7* (CFQ-7).

		*a*DIF			*b*DIF	
Item	*χ*^2^	*df*	*p*	*χ*^2^	*df*	*p*
**1**	.9	1	.34	4.6	6	.59
**2**	1.1	1	.29	13.6	6	.03
**3**	.1	1	.78	8.8	6	.19
**4**	.4	1	.51	6.5	6	.37
**5**	.0	1	.83	9.4	6	.15
**6**	.4	1	.54	5.3	6	.51
**7**	.0	1	.91	3.9	6	.69

After verifying invariance, we explored the differences in CFQ-7 total scores across the samples. There was a significant difference between the non-clinical (*M* = 25.50, *SD* = 8.55) and the clinical group (*M* = 32.38, *SD* = 7.23), the latter showing significantly higher values (*t* (363) = -7.32, *p* < .001, Cohen’s *d* = .87).

## Discussion

It is increasingly recognized that low PF is a transdiagnostic factor for psychopathology [1–4]. CF is one of the core processes contributing to a poorer PF and, hence, to many mental health conditions [2, 6]. The CFQ-7 is a widely used tool for measuring CF [10, 11]. However, the psychometric properties of the scale have been addressed only by means of the CTT, and few studies have explored its measurement invariance across non-clinical and clinical groups, with findings that are partly contradictory [10, 14, 22]. Hence, the first aim of the present study was to investigate the characteristics of the items and the criterion validity of the scale using the IRT, after confirming the unidimensionality of the CFQ-7 in a non-clinical sample.

In line with previous research [10, 12, 15, 18, 21, 22], confirmatory factor analyses supported the unidimensionality of the scale. Furthermore, all item loadings were high, suggesting that all the items contribute highly to measuring CF. The results of IRT analyses showed that CFQ-7 items assess a wide range of CF severity among non-clinical subjects and that they are useful to discriminate different levels of CF. Moreover, the results indicated that the scale is sufficiently informative for a broad range of the trait. Thus, with respect to previous psychometric studies on the CFQ-7, conducted through the CTT, the current IRT analysis allowed us to understand that this instrument is able to adequately measure both low and high levels of the trait. Thus, the instrument can be applied both for screening and for clinical purposes.

We also examined the criterion validity of the CFQ-7 by testing the relationships of CF with theoretically related constructs and outcomes. As expected, higher CF was associated with higher levels of depression and poorer life satisfaction. The correlations of committed action with both depression and life satisfaction were also in the theoretically expected direction. Furthermore, the results showed that higher CF significantly related to lower committed action, supporting that CF and committed action are two interrelated processes. These results are in line with theoretical predictions in the ACT model as well as with the growing body of findings linking PF processes to a broad range of mental health-related outcomes [12, 31–33, 40, 64–66], and they provide further support to the criterion validity of the CFQ-7.

The second aim of this study was to investigate measurement invariance of the CFQ-7 across non-clinical and clinical subjects using an IRT framework. In this regard, only a few previous studies have investigated measurement invariance of the CFQ-7 across non-clinical and clinical samples, and all of them were conducted in a CTT framework and provided partially contrasting findings [10, 14, 22]. In this study we conducted DIF analyses, considered to be one of the most efficient methods of analyzing a test's measurement invariance [37], to investigate whether individuals with the same level of the trait, but from different groups, differ in the probability of answering the CFQ items similarly. In this study, no item showed DIF, hence proving that the CFQ-7 is invariant across population types. Thus, the findings in this study suggest that the CFQ-7 can be used in both clinical and non-clinical samples. With respect to previous studies on the CFQ-7 conducted by applying CTT, this IRT investigation brings new information about measurement invariance as it has been conducted at the item level and across respondents belonging to different groups but having the same level of the trait.

Furthermore, measurement invariance of the CFQ-7 provides the possibility to interpret differences in CF across these kinds of samples as true differences in the variable. Hence, after verifying measurement invariance of the CFQ-7 across samples, we investigated between-group differences in CF. Students in the clinical group yielded higher CF than those in the non-clinical group. These results are consistent with the growing body of research suggesting that CF constitutes a core variable affecting students’ well-being [e.g., 12, 21, 22]. Clinical subjects in this study were undergraduate students with mild to moderate levels of psychological distress. It is worth noting that approximately half of university students report significant levels of psychological distress (mainly in the form of anxiety and depression phenomenology), which in turn can negatively impact different areas of functioning, including academic performance [67, 68]. These and other related findings in clinical psychology have led to an increasing emphasis on developing transdiagnostic models, such as the ACT model, that can be applied to a range of mental health issues [4]. Overall, findings from this study bring additional evidence of the applicability of the ACT model in the study of psychological distress and life satisfaction among college students.

In this context, a brief and general measure of CF, such as the CFQ-7, represents a relevant instrument to further investigate the relationships of PF components with mental health in a wide range of settings. Moreover, the CFQ-7 may be used in clinical settings at pre-treatment to assess whether CF constitutes one of the processes to be taken care of. It may also be used during the treatment phase to explore the mechanisms through which psychological interventions affect outcome measures [10]. In this regard, there is evidence showing that clinical improvements obtained with ACT-based interventions are accounted for by changes in PF processes, including changes in CF [10, 14, 69, 70]. Hence, ACT-based interventions constitute a promising approach to address psychological processes that negatively impact mental health in college students, such as CF [4].

Despite these promising results, this study has some limitations. Psychological problems in the clinical group were not classified using the Diagnostic and Statistical Manual for Mental Disorders-5 (DSM-5) [71] or the International Classification of Diseases-10 (ICD-10) [72]. In this study, measurement invariance of the CFQ-7 has been explored in a specific sample of undergraduate students with mild to moderate anxiety and/or depression symptoms. Thus, future research could evaluate measurement invariance of this scale in other psychological clinical samples. Moreover, the samples involved in this study consisted of young adults only, with more females than males. Future studies could include samples with a wider age range and better gender balance in order to analyze DIF across both age and gender. In addition, even though the internal consistency of the CAQ proved to be adequate in our non-clinical group, the CAQ has not been specifically validated in Italian samples. It should also be highlighted that non-clinical participants in this study completed a paper-and-pencil version of the assessment protocol, while clinical participants completed a web-based version of the CFQ-7. There are concerns about the quality of data from web-based surveys as well as about the psychometric equivalence between web- and paper-based methods [73]. Hence, future research could also explore whether the data collection method affects responses to the CFQ-7.

## Supporting information

S1 Appendix Italian version of the Cognitive Fusion Questionnaire-7 (CFQ-7). (DOCX)

S1 Fig The Response Characteristics Curve (RCC) of each CFQ-7 item. The horizontal axis shows the latent trait (Theta); the vertical axis shows the probability of selecting each response option at a given level of the trait.
(DOCX)

S1 Dataset Dataset for the clinical and non-clinical sample. (SAV)
