# Supplementary material for: Measuring cognitive fusion through the Cognitive Fusion Questionnaire-7: Measurement invariance across non-clinical and clinical psychological samples
Source: PLoS One. 2021 Feb 3;16(2):e0246434. doi: 10.1371/journal.pone.0246434 (PMC7857615; doi:10.1371/journal.pone.0246434)
Supplement: S1 Appendix — (DOCX) [file pone.0246434.s001.docx]

**S1 Appendix. Italian version of the Cognitive Fusion Questionnaire – 7 (CFQ-7)**

|  | **Item** | **non è mai vero** | **è vero molto raramente** | **è vero raramente** | **è vero a volte** | **è spesso vero** | **è vero quasi sempre** | **è sempre vero** |
| --- | --- | --- | --- | --- | --- | --- | --- | --- |
|  | I miei pensieri mi causano angoscia e sofferenza emotiva | 1 | 2 | 3 | 4 | 5 | 6 | 7 |
|  | Sono così preso dai miei pensieri che non riesco a fare ciò che più desidero fare | 1 | 2 | 3 | 4 | 5 | 6 | 7 |
|  | Analizzo troppo le situazioni, al punto tale che non mi è più utile | 1 | 2 | 3 | 4 | 5 | 6 | 7 |
|  | Lotto contro i miei pensieri | 1 | 2 | 3 | 4 | 5 | 6 | 7 |
|  | Mi arrabbio con me stesso per avere certi pensieri | 1 | 2 | 3 | 4 | 5 | 6 | 7 |
|  | Tendo a rimanere invischiato nei miei pensieri | 1 | 2 | 3 | 4 | 5 | 6 | 7 |
|  | È davvero faticoso lasciare andare i pensieri che mi turbano, anche se so che lasciarli andare sarebbe utile | 1 | 2 | 3 | 4 | 5 | 6 | 7 |
