# Supplementary material for: Measuring cognitive fusion through the Cognitive Fusion Questionnaire-7: Measurement invariance across non-clinical and clinical psychological samples
Source: PLoS One. 2021 Feb 3;16(2):e0246434. doi: 10.1371/journal.pone.0246434 (PMC7857615; doi:10.1371/journal.pone.0246434)
Supplement: S1 Fig — The horizontal axis shows the latent trait (Theta); the vertical axis shows the probability of selecting each response option at a given level of the trait. (DOCX) [file pone.0246434.s002.docx]

**S1. Fig. The item characteristics curve (ICC) of each CFQ-7 item.**

|  |  |  |  |
| --- | --- | --- | --- |
|  |  |  |  |

Latent trait (Theta) is shown on the horizontal axis and the probability of selecting a particular response option at a given level of the latent trait is shown on the vertical axis.
